# Associations between selective attention and soil-transmitted helminth infections, socioeconomic status, and physical fitness in disadvantaged children in Port Elizabeth, South Africa: An observational study

**DOI:** 10.1371/journal.pntd.0005573

**Published:** 2017-05-08

**Authors:** Stefanie Gall, Ivan Müller, Cheryl Walter, Harald Seelig, Liana Steenkamp, Uwe Pühse, Rosa du Randt, Danielle Smith, Larissa Adams, Siphesihle Nqweniso, Peiling Yap, Sebastian Ludyga, Peter Steinmann, Jürg Utzinger, Markus Gerber

**Affiliations:** 1 Department of Sport, Exercise and Health, University of Basel, Basel, Switzerland; 2 Swiss Tropical and Public Health Institute, Basel, Switzerland; 3 University of Basel, Basel, Switzerland; 4 Department of Human Movement Science, Nelson Mandela Metropolitan University, Port Elizabeth, South Africa; 5 Department of HIV&AIDS Research, Nelson Mandela Metropolitan University, Port Elizabeth, South Africa; 6 Institute of Infectious Disease and Epidemiology, Tan Tock Seng Hospital, Singapore; Universidad Nacional Autónoma de México, MEXICO

## Abstract

**Background:**

Socioeconomically deprived children are at increased risk of ill-health associated with sedentary behavior, malnutrition, and helminth infection. The resulting reduced physical fitness, growth retardation, and impaired cognitive abilities may impede children’s capacity to pay attention. The present study examines how socioeconomic status (SES), parasitic worm infections, stunting, food insecurity, and physical fitness are associated with selective attention and academic achievement in school-aged children.

**Methodology:**

The study cohort included 835 children, aged 8–12 years, from eight primary schools in socioeconomically disadvantaged neighborhoods of Port Elizabeth, South Africa. The d2-test was utilized to assess selective attention. This is a paper and pencil letter-cancellation test consisting of randomly mixed letters *d* and *p* with one to four single and/or double quotation marks either over and/or under each letter. Children were invited to mark only the letters *d* that have double quotation marks. Cardiorespiratory fitness was assessed via the 20 m shuttle run test and muscle strength using the grip strength test. The Kato-Katz thick smear technique was employed to detect helminth eggs in stool samples. SES and food insecurity were determined with a pre-tested questionnaire, while end of year school results were used as an indicator of academic achievement.

**Principal findings:**

Children infected with soil-transmitted helminths had lower selective attention, lower school grades (academic achievement scores), and lower grip strength (all *p*<0.05). In a multiple regression model, low selective attention was associated with soil-transmitted helminth infection (*p*<0.05) and low shuttle run performance (*p*<0.001), whereas higher academic achievement was observed in children without soil-transmitted helminth infection (*p*<0.001) and with higher shuttle run performance (*p*<0.05).

**Conclusions/Significance:**

Soil-transmitted helminth infections and low physical fitness appear to hamper children’s capacity to pay attention and thereby impede their academic performance. Poor academic achievement will make it difficult for children to realize their full potential, perpetuating a vicious cycle of poverty and poor health.

**Trial registration:**

ClinicalTrials.gov ISRCTN68411960

## Author summary

Children growing up in challenging environments, such as townships in South Africa, are at an increased risk of ill-health associated with sedentary behavior, poor nutrition, growth retardation, and infections with parasitic worms. Negative factors such as limited educational resources, insufficient health care and safety are exacerbating the effects of poverty and, taken together, might cause developmental delays and school failure. A total of 835 school children aged 8–12 years were examined for soil-transmitted helminth infection, physical fitness, selective attention, stunting, household socioeconomic conditions, and food security. Furthermore, children’s academic achievement scores were utilized as a proxy for academic achievement. The multivariate analyses showed that low selective attention was associated with soil-transmitted helminth infection and low shuttle run performance, whereas higher academic achievement was observed in children without soil-transmitted helminth infection and with higher shuttle run performance. Our study suggests that soil-transmitted helminths and low physical fitness hinder children from realizing their full potential.

## Introduction

Attention skills are relevant for academic foundations and are important for learning [1]. Selective attention is the ability to select and focus on a particular task, while simultaneously suppressing irrelevant or distracting information. Competing information can occur both externally and internally due to visual or auditory distractions or distracting thoughts [2]. Selective attention has been associated with important domains in education, such as language processing [3], literacy [4], and numeracy [5], and hence, plays an important role in academic achievement. A growing body of literature documents that children from low-income households exhibit more attention deficits compared to their higher-income peers [6,7]. Of note, academic achievement depends on multiple factors such as educational opportunity, socioeconomic status (SES), health and nutritional status, family environment [8,9], social competence [10], cognitive skills, and the ability to pay attention [2].

Children growing up in socioeconomically deprived environments face multiple challenges. Essential services, such as health care, sanitation, physical security, electricity, and high quality academic and physical education are often lacking, with serious consequences for children’s psychological and physiological development and wellbeing [11]. Poverty also limits the parents’ ability to provide a responsive, supportive, and safe learning environment [12], and lessens the probability that children will have access to cognitively stimulating materials (e.g., books and toys) [13]. Families with low income often invest most of their resources into covering their basic needs, such as food and housing, and have therefore limited means to invest in the future of their children [14]. Poverty also puts children at risk of chronic malnutrition [14]. Chronic malnutrition causes stunting and has been found to be associated with poor cognitive development resulting in low IQ, and problems with motor development [15]. This, in turn, can impede children’s ability to concentrate, process information, and focus on academic work [16].

Poor living conditions with a lack of clean water, inadequate sanitation, and insufficient hygiene also favor parasitic worm and intestinal protozoa infections [17,18], which may lead to symptoms such as abdominal pain, diarrhea, anemia, growth retardation, reduced physical fitness, cognitive impairment, and poor academic achievement [19,20]. Recent systematic reviews suggest associations between parasitic worm infection and children’s cognitive function and academic performance, but positive effects of mass treatment on cognition or school performance remain elusive [21–24]. A study by Ezeamama et al. [25] found that roundworm (*Ascaris lumbricoides*) infection was associated with poor performance on tests of memory, whereas whipworm (*Trichuris trichiura*) infection was associated with poor performance on tests of verbal fluency among Filipino children. To our knowledge, there is a paucity of studies investigating whether soil-transmitted helminth infections are associated with selective attention.

Children from families with low SES are also less likely to have access to health care or health insurance, resulting in a greater risk of illness and school absenteeism and consequently a lack of academic input compared to better-off peers [16]. Recent reviews and meta-analyses have shown that physical activity elicits short- and long-term benefits for children’s executive function [26], attention [27], and other academic outcomes [28]. Yet, physical activity levels are often low among poor children and youth, also in South Africa [29]. For instance, a study by Walter [30], which focused on primary school children in disadvantaged schools, observed that most children do not achieve the recommended 60 min of daily moderate-to-vigorous physical activity (MVPA). These results are not surprising given that sport and recreation facilities are often inadequate, inaccessible, or in poor condition, while qualified teachers are scarce and physical education and extramural sport programs are rare [31].

The purpose of the present study was to find out how children’s selective attention and academic achievement relate to age, sex, SES, helminth infection status, stunting, food security, and physical fitness. In a first step, we looked at bivariate associations and compared children with or without helminth infection, and stunted or non-stunted children. In a second step, we examined multivariate associations to find out how age, sex, SES, helminth infection status, stunting, food security, and physical fitness relate to selective attention and academic achievement if all these variables are considered simultaneously.

## Methods

### Ethics statement

The “Disease, Activity and School children’s Health” (DASH) cohort study was approved by the ethical review board of Northwestern and Central Switzerland (EKNZ; reference no. 2014–179, approval date: 17 June 2014), the Nelson Mandela Metropolitan University (NMMU) Human Ethics Committee (study number H14-HEA-HMS002, approval date: 4 July 2014), the Eastern Cape Department of Education (approval date: 3 August 2014), and the Eastern Cape Department of Health (approval date: 7 November 2014) in Port Elizabeth, South Africa. The study is registered at ISRCTN registry under controlled-trials.com (unique identifier: ISRCTN68411960, registration date: 1 October 2014).

Details regarding the information of potential study participants, exclusions due to medical reasons, management of helminth infections, and referrals, are provided in a previously published study protocol [32]. In brief, oral assent from each participating child was sought and individual written informed consent was obtained from parents/guardians. Participation was voluntary and children could withdraw from the study at any time without further obligations. Children were eligible for this study if they met the following inclusion criteria: (i) are willing to participate in the study; (ii) have a written informed consent by a parent/guardian; (iii) are not participating in other clinical trials during the study period; and (iv) do not suffer from medical conditions, which will prevent participation in the study, as determined by qualified medical personnel. To ensure confidentiality, each study participant was given a unique identification number. All tests were available in English, Xhosa, and Afrikaans. To ensure optimal translation of the tests, we collaborated with independent professional translators and followed the procedure set out by Brislin [33]. Thus, test instructions and items were translated from English into Xhosa and Afrikaans, and pilot-tested with a small sample of Xhosa and Afrikaans speaking students and school children of the same age as the study cohort. Schools were recruited from 2014 to 2015. Data assessment took place between February 2015 and March 2015.

### Study population and procedures

The study involved 8- to 12-year-old children attending grade 4 from eight schools located in socioeconomically disadvantaged neighborhoods in Port Elizabeth, South Africa. South African public schools are classified into five groups, with quintile one standing for the poorest and quintile five for the least poor [34]. Study schools belonged to quintile three. The sample size calculation for the study was based on achieving sufficient precision in estimating the prevalence of soil-transmitted helminth infections, with a targeted sample size of approximately 1000 grade 4 school children (for more details regarding power calculation see Yap et al. [32]).

### Body weight and height

All children were asked to remove their shoes and jerseys/jackets before standing on a digital weighing scale (Micro T7E electronic platform scale, Optima Electronics; George, South Africa). Body weight was measured once and recorded to the nearest 0.1 kg. With the shoes removed, each child then stood against a Seca stadiometer (Surgical SA; Johannesburg, South Africa) with their back erect and shoulders relaxed. Body height was measured once and recorded to the nearest 0.1 cm.

### Physical fitness

Upper body strength was determined by the grip strength test [35]. The Saehan hydraulic hand dynamometer (MSD Europe BVBA; Tisselt, Belgium) was employed. The field investigator demonstrated how to hold the hand dynamometer and instructed the child to sit relaxed, spine erect, and arm position at a 90° angle. Each child had six trials, alternating between the right and left hand with a 30 sec resting period between trials, griping the hand dynamometer as hard as possible. All six trials were recorded to the nearest 1 kg and averaged.

To measure children’s aerobic fitness, the 20 m shuttle run test was utilized, following the test protocol described by Léger et al. [36]. A premeasured running course was laid out on a flat terrain and marked with color-coded cones. Children who felt sick or voiced discomfort were excluded. The test procedures were explained and a researcher demonstrated two trial runs. Once children were familiar with the test procedures, they started with a running speed of 8.5 km/h, following a researcher who set the pace according to the acoustic signal. The frequency of the sound signal gradually increased every min by 0.5 km/h. If a child was unable to cross the marked 2 m line before each end of the course at the moment of the sound signal for two successive intervals, the individual maximum was reached. Children were then asked to stop running and the fully completed laps were noted.

### Socioeconomic status

To estimate children’s SES, they were asked to answer nine items, covering household-level living standards, such as infrastructure and housing characteristics (house type, number of bedrooms, type of toilet and access to indoor water, indoor toilet/bathroom, and electricity) and questions related to ownership of three durable assets (presence of a working refrigerator, washing machine, and car). The dichotomized items (0 = poor quality, not available; 1 = higher quality, available) were summed up to build an overall SES index, with higher scores reflecting higher SES. The validity of similar measures has been established in previous research [37].

### Food insecurity

Food insecurity was measured with four questions about hunger, portion size, and meal frequency (e.g., “did you go to bed hungry last night?”). The items were adapted from the Household Hunger Scale [38]. Response options were summed up to obtain a score for each participant ranging from 0 (food insecure/hungry) to 4 (food secure/not hungry). This score was used to obtain an overall index of food security, with higher scores reflecting higher food security.

### Helminth infections

To diagnose helminth infections, stool containers with unique identifiers were handed out to school children with the instruction to return them with a small portion of their own morning stool. The diagnostic work-up was done on the same day. Duplicate 41.7 mg Kato-Katz thick smears were prepared from each stool sample [39]. Slides were independently read under a microscope by experienced laboratory technicians who counted the number of helminth eggs and recorded them for each species separately. For quality control, a random sample of 10% of all Kato-Katz thick smears was re-examined by a senior technician. In case of discordant results, the slides were re-read a third time and results discussed among the technicians until agreement was reached. Soil-transmitted helminth egg counts were multiplied by a factor of 24 to obtain a proxy for helminth infection intensity, as expressed by the number of eggs per 1 g of stool (EPG) [40]. Subsequently, a single 400 mg oral dose of albendazole (INRESA; Bartenheim, France) was administered to all children participating in the study, according to WHO and national treatment guidelines. Otherwise, to our knowledge, no further helminthiasis control interventions took place in recent years in the study community where the cohort group stems from.

### Selective attention and academic achievement

Children’s selective attention capacity was measured with the d2 attention test, developed by Brickenkamp et al. [41]. The d2 test determines the capacity to focus on one stimulus/fact, while suppressing awareness to competing distractors. The d2 attention test is a paper and pencil letter-cancellation test that consists of 14 lines of 47 randomly mixed letters *d* and *p*. Participants were instructed to identify and mark all *d* letters with two dashes arranged either as single dashes (i.e., one above and one below the *d*s), or in pairs above or below the *d*s. After 20 sec, the researcher signaled to continue on the next line. Altogether, the test lasted 4 min and 40 sec. The test was performed in groups of 20–25 students and conducted during the first school lesson in a quiet room, with an average room temperature of 24°C. Pencils were distributed and the test procedure was explained to the children in their native language. Additionally, a practice line was provided on the blackboard to ensure that all participants understood the test procedures. Furthermore, children were encouraged to practice on the test line prior to launching the test.

As shown in Table 1, several different parameters can be calculated after completion of the d2 test. For instance, the total number of items processed is a measure of processing speed (TN), while the number of all errors relative to the total number of items processed is a measure of precision and thoroughness, referred to as accuracy in the present text (E%). By contrast, the number of correctly marked characters minus the number of incorrectly marked characters is a measure of concentration ability and performance (CP). E% and CP are not inflated by excessive skipping as they are based on the number of target and non-target characters cancelled as opposed to processing speed which can be influenced by test strategies [42]. In our study, we therefore used E% and CP as dependent variables due to their resistance to falsification. Processes of selective attention are required for successful completion since not only the letter *d* is orthographically similar to the letter *p*, but there are many distracting letters with more or less than two dashes [41].

**Table 1:** Abbreviations, descriptions, and calculation of d2 test of attention.

d2 test measures	Acronym	Description of measure	Computation
Processing speed	TN*	Total number of characters processed	Sum of number of characters processed before the final cancellation on each trial
Processing speed	TN-E*	Total correctly processed	Total characters processed minus total errors made
Inattention	O*	Errors of omission	Sum of number of target symbols not cancelled
Impulsivity	C*	Errors of commission	Sum of number of non-targets symbols cancelled
Accuracy	E%*	Percentage of errors	Total number of errors divided by the total number of characters processed
Concentration performance	CP*	Concentration performance	Total number of correctly cancelled minus total number incorrectly cancelled

*Notes*. Abbreviations* designated in the d2 manual [41]

As an indicator of academic achievement, we collected from each school the children’s end-of-year results which are based on the mean of four subjects: (i) home language (Xhosa or Afrikaans, in this case); (ii) first additional language (English, in this case); (iii) mathematics; and (iv) life skills. Learner achievement in each subject is graded on a scale of 1 to 7, whereby a rating of 1 (0–29%) indicates “not achieved” and one of 7 (80–100%) indicates “outstanding achievement”. A rating of 4 (50–59%) indicates “adequate achievement”.

### Statistical analysis

Data were double-entered, validated using EpiData version 3.1 (EpiData Association; Odense, Denmark), and merged into a single database. Statistical analyses were performed with SPSS version 23 (IBM Corporation; Armonk, United States of America) for Windows and STATA version 13.0 (STATA; College Station, United States of America). Anthropometric indicators and fitness performance scores were expressed as means (*M*) and standard deviations (*SD*). To describe the anthropometry of the children, body weight and height values were utilized to calculate the body mass index (BMI), defined as weight (in kg)/height^2^ (in m^2^). BMI-for-age and height-for-age (HAZ) were thus available for every participant [43]. The BMI and height-for-age z-scores (HAZ) were calculated using the World Health Organization (WHO) growth reference [43]. The sex-adjusted HAZ z-scores were used as an indicator for stunting [44]. The level at which the child stopped running during the 20 m shuttle run test was used to calculate an estimate of maximal oxygen uptake (VO_2_ max), readily adjusted for age [36]. The parasitological status was expressed in terms of prevalence of helminth infection. Selective attention was expressed as raw values. Statistical significance was set at *p*<0.05.

In a first step, separate mixed linear and mixed logistic regression models with random intercepts for school classes were calculated to compare selective attention and physical fitness among (i) stunted and normally grown children; and (ii) soil-transmitted helminth infected and non-infected children. In a second step, SES, age, sex, soil-transmitted helminth infection status, stunting, food insecurity, grip strength, and VO_2_ max were analyzed simultaneously in multiple linear regression models, with random intercepts for school classes, in order to determine the simultaneous impact of these variables on selective attention and academic achievement. To interpret the findings, the following statistical coefficients were displayed: for mixed linear and mixed logistic regression models the means and 95% confidence interval (CI), and for multiple linear regression models the unstandardized B coefficients in combination with the 95% CI.

## Results

### Study population

**Fig 1:**
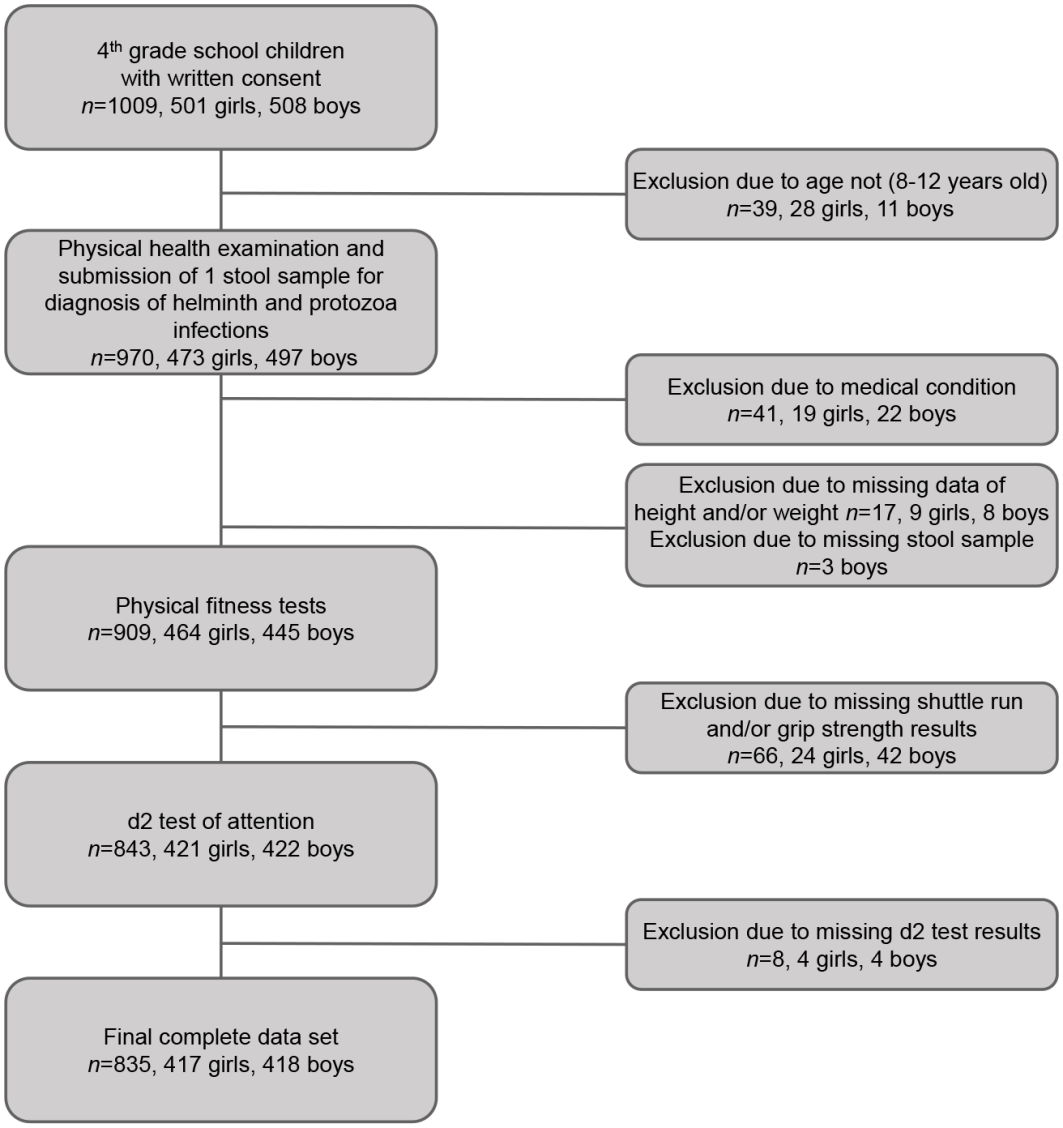
Participant flow chart diagram.

As shown in the participant flow chart diagram (Fig 1), after receiving written informed consent from a parent or legal guardian, a total of 1,009 students agreed to take part in the study. Data of 970 children were available for further analyses. Complete data records were available for 835 children; 61.8% (n = 516) were black African (mostly Xhosa speaking), while the remaining 38.2% (n = 319) were colored African (mostly Afrikaans speaking). All analyses presented in this article refer to this final cohort, including 417 girls (49.9%) and 418 boys (50.1%).

### Anthropometric indicators, helminth infection, stunting, food insecurity, and SES

An overview of the descriptive statistics and sex differences for all study variables is provided in Table 2. Boys were, on average, slightly older than girls and had a lower BMI. Overall, 31.0% of the children were infected with *T*. *trichiura* and/or *A*. *lumbricoides*, yet no hookworm infections were found. Stunting was observed in 12.3% of the children and the mean food insecurity score was 3.1. No significant sex differences were identified for height, weight, helminth infection status, stunting, food insecurity, and SES. Stratification by age (Table 3) revealed that older children were significantly taller, heavier, and more stunted, and had a higher prevalence of helminth infection.

**Table 2:** Characteristics of the study population, stratified by sex, based on mixed linear and mixed logistic regression analyses.

	Total (*n* = 835)		Males (*n* = 418)		Females (*n* = 417)		*p*-value^a^
Parameter	*M* (*SD*)		*M* (*SD*)		*M* (*SD*)		
**Age and anthropometry**							
Age (years)	9.5 (0.9)		9.7 (0.9)		9.4 (0.9)		**<0.001**
Height (cm)	133.2 (7.1)		133.3 (6.7)		133.1 (7.5)		0.584
Weight (kg)	30.5 (7.5)		30.0 (6.5)		31.0 (8.2)		0.087
BMI (kg/m^2^)	17.0 (3.1)		16.8 (2.6)		17.3 (3.3)		**0.015**
**Physical fitness**							
VO_2_ max^b^ (in ml kg^-1^ min^-1^)	49.1 (4.3)		50.8 (4.3)		47.3 (3.5)		**<0.001**
Grip strength^c^ (in kg)	12.0 (3.1)		12.7 (3.1)		11.4 (2.9)		**<0.001**
**Selective attention**							
E%^d^	16.8 (12.9)		16.4 (13.3)		17.1 (12.4)		0.397
CP^e^	54.5 (30.5)		53.9 (29.9)		55.0 (31.2)		0.486
**Academic achievement**							
Academic achievement scores^f^	4.3 (1.5)		4.0 (1.6)		4.5 (1.4)		**<0.001**
**Sociocultural characteristics**							
Socioeconomic status (SES)^g^	7.2 (2.0)		7.2 (2.0)		7.3 (2.0)		0.778
Food security^h^	3.1 (0.9)		3.1 (0.9)		3.1 (0.9)		0.482
**Prevalence of helminth infection and stunting**		n (%)		n (%)		n (%)	
Infected^i^		333 (31.1)		182 (32.5)		151 (29.6)	0.462
Double infection^j^		135 (16.2)		75 (17.9)		60 (14.4)	0.089
Stunted^k^		103 (12.3)		50 (12.0)		53 (12.7)	0.535

All statistically significant differences are marked in bold.

^a^All *p*-values are calculated using either mixed linear or mixed logistic regression, as appropriate, adjusted for clustering of school classes.

^b^All mean VO_2_ estimates are expressed in ml kg^-1^ min^-1^ and adjusted for age.

^c^All mean grip strength values are expressed in kg and are not adjusted for age.

^d^E% = Percent of errors.

^e^CP = Concentration performance.

^f^Academic achievement scores: Average of the four subjects Home Language, Additional Language, Mathematics and Life Skills (n = 777)

^g^SES: Socioeconomic status measured by ownership and housing-related questions on a scale from 0 to 9 points (0 = low score).

^h^Food security measured with the hunger scale ranging from 0 to 4 (0 = hungry/food insecure, 4 = not hungry/food secure).

^i^Infected with one or two soil-transmitted helminth species (*A*. *lumbricoides* and/or *T*. *trichiura*).

^j^Double infection: Infected with two soil-transmitted helminth species (*A*. *lumbricoides* and *T*. *trichiura*).

^k^Stunting is defined as <-2HAZ.

**Table 3:** Characteristics of the study population, stratified by age and expressed as means and 95% CI or %, and differences between age groups based on mixed linear and mixed logistic regression analyses.

Age (years)	8 (*n* = 76) *M* (95% CI) or %	9 (*n* = 387) *M* (95% CI) or %	10 (*n* = 245) *M* (95% CI) or %	11 (*n* = 108) *M* (95% CI) or %	12 (*n* = 19) *M* (95% CI) or %	*p*-value^h^
**Anthropometry**						
Height (cm)	130.5 (129.1–131.9)	131.3 (130.7–131.9)	134.4 (133.5–135.3)	137.7 (136.1–139.2)	139.9 (137.5–142.2)	**<0.001**
Stunted (%)^a^	2.6	6.7	16.3	25.0	42.1	**<0.001**
Weight (kg)	29.7 (28.2–31.1)	29.8 (29.0–30.5)	31.0 (30.0–31.9)	32.2 (30.8–33.6)	32.8 (30.5–35.1)	**<0.001**
BMI (kg/m^2^)	17.3 (16.7–18.0)	17.1 (16.8–17.4)	17.0 (16.6–17.3)	16.8 (16.3–17.3)	16.7 (15.8–17.6)	0.675
**Sociocultural characteristics**						
Socioeconomic status (SES)^b^	7.9 (7.6–8.3)	7.5 (7.4–7.7)	6.8 (6.6–7.1)	6.6 (6.2–7.1)	6.8 (5.6–8.1)	**<0.001**
Food security^c^	3.1 (2.9–3.4)	3.1 (3.0–3.2)	3.1 (3.0–3.4)	3.2 (3.0–3.4)	3.0 (2.6–3.3)	0.959
Infected (%)^d^	5.3	23.3	41.2	51.9	42.1	**0.007**
**Physical fitness**						
VO_2_ max (in ml kg^-1^ min^-1^)^e^	50.3 (49.5–51.1)	49.1 (48.7–49.5)	48.8 (48.2–49.3)	48.6 (47.5–49.6)	48.6 (46.0–51.3)	**0.014**
Grip strength (in kg)^f^	11.1 (10.4–11.8)	11.4 (11.1–11.7)	12.5 (12.1–12.8)	13.7 (13.1–14.4)	14.3 (12.9–15.6)	**<0.001**
**Selective attention**						
E% (percentage of errors)	14.4 (11.9–16.9)	15.8 (14.5–17.0)	16.8 (15.2–18.5)	21.1 (18.5–23.7)	20.3 (13.9–26.8)	**<0.001**
CP (concentration performance)	57.6 (52.0–63.1)	57.1 (54.2–60.0)	52.4 (48.5–56.3)	48.9 (42.1–55.8)	47.1 (29.8–64.3)	0.071
**Academic achievement**						
Academic achievement scores^g^	5.2 (4.9–5.8)	4.8 (4.7–5.0)	3.8 (3.6–3.9)	3.0 (2.8–3.3)	2.7 (2.0–3.3)	**<0.001**

*Notes*. All statistically significant coefficients are marked in bold.

^a^Stunted: is defined as <-2HAZ.

^b^SES: Socioeconomic status measured by ownership and housing-related questions on a scale from 0–9 points (0 = low score).

^c^Food security measured with the hunger scale ranging from 0–4 (0 = hungry/food insecure, 4 = not hungry/food secure).

^d^Infected with one or two soil-transmitted helminth species (*A*. *lumbricoides* and/or *T*. *trichiura*).

^e^All mean VO_2_ estimates are expressed in ml kg^-1^ min^-1^ and are adjusted for age.

^f^All mean grip strength values are expressed in kg and are not adjusted for age.

^g^Academic achievement scores: Average of the four subjects Home Language, Additional Language, Mathematics and Life Skills, n = 777.

^h^All *p*-values are calculated using either mixed linear or mixed logistic regression, adjusted for clustering within school classes.

### Physical fitness, stratified by sex and age

As shown in Table 2, boys achieved significantly higher mean grip strength and had a higher mean VO_2_ max estimate than girls. As shown in Table 3, older children (aged 10–12 years) achieved higher mean grip strength scores than their younger peers (8- to 9-years old). The stratification by age also revealed that the younger group (8–9 years) reached a higher estimated VO_2_ max than the older group (10–12 years).

### Selective attention, stratified by sex and age

As displayed in Table 2, stratification by sex revealed that girls and boys did not differ with regard to their selective attention capacity. Younger children had a significantly lower percentage of errors (see Table 3 for mean scores).

### Academic achievement, stratified by sex and age

Girls reached statistically significantly higher academic scores than boys (Table 2). Stratification by age revealed that older children’s academic achievement was lower than younger children’s academic achievement (see Table 3 for mean scores). A higher percentage of errors in the attention test was associated with poorer academic achievement (*r* = -0.33, *p*<0.05), whereas a positive association was observed between concentration performance (CP) and academic achievement (*r* = 0.33, *p*<0.05), as assessed by students’ academic achievement scores.

### Association of soil-transmitted helminth infection status and stunting with physical fitness, selective attention, and academic achievement

As shown in Table 4, children with no soil-transmitted helminth infection had higher mean grip strength test results compared to their infected counterparts. The comparison between stunted and non-stunted children revealed that children not classified as being stunted achieved significantly higher mean grip strength test results. The mean VO_2_ max results did not differ between the two groups.

**Table 4:** Physical fitness, stratified by soil-transmitted helminth infection and stunting (means and 95% CI); between-group differences based on mixed linear and mixed logistic regression analyses.

	Non-infected (*n* = 576) *M* (95% CI)	Infected^d^ (*n* = 259) *M* (95% CI)	*p*-value^c^	Non-stunted (n = 732) *M* (95% CI)	Stunted^e^ (n = 103) *M* (95% CI)	*p*-value^c^
**Fitness**						
VO_2_ max (in ml kg^-1^ min^-1^)^a^	49.3 (48.9–49.6)	48.6 (48.1–49.1)	0.149	49.0 (48.7–49.4)	49.1 (48.2–49.9)	0.664
Grip strength (in kg)^b^	12.4 (12.2–12.7)	11.2 (10.9–11.54)	**0.013**	12.3 (12.1–12.5)	10.2 (9.7–10.7)	**<0.001**

*Notes*. All statistically significant coefficients are marked in bold.

^a^All mean VO_2_ max estimates are expressed in ml kg^-1^ min^-1^ and are adjusted for age.

^b^All mean grip strength values are expressed in kg and are not adjusted for age.

^c^All *p*-values are calculated using either mixed linear or mixed logistic regression, adjusted for clustering within school classes.

^d^Infected with one or two soil-transmitted helminth species (*A*. *lumbricoides* and/or *T*. *trichiura*).

^e^Stunted: is defined as <-2 HAZ.

**Fig 2:**
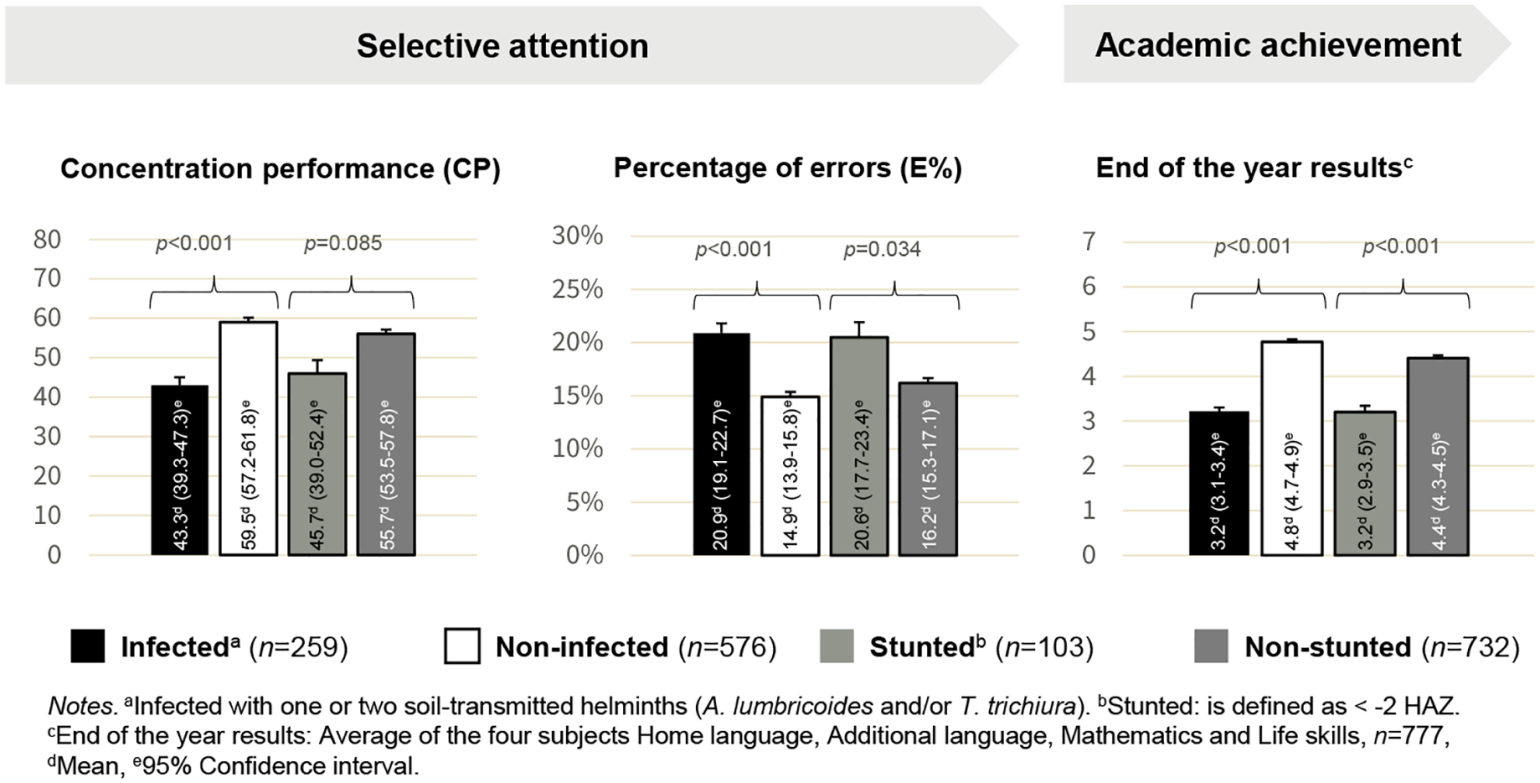
Group differences between infected and non-infected children and stunted and non-stunted children in their concentration performance, percentage of errors, and academic achievement scores.

Fig 2 shows the univariate comparisons between infected versus non-infected and stunted versus non-stunted children in selective attention and academic performance. As illustrated, in these uncontrolled analyses, children infected with soil-transmitted helminths performed weaker on the d2 test of attention, compared to their non-infected counterparts. Stunted children had a lower mean concentration performance and a higher mean percentage of errors, but only the latter was statistically significant. Children without a soil-transmitted helminth infection and non-stunted children achieved statistically significantly higher academic achievement scores compared to their infected and stunted peers. Additional analyses showed that infected children had a significantly higher risk of being stunted, and *vice versa* (infected children: 25% stunted; non-infected children: 7% stunted; stunted children: 62% infected; non-stunted children: 27% infected χ^2^[1,835] = 53.2, *p* < .001). Accordingly, multivariate analyses were performed in the next step to avoid problems associated with multi-collinearity.

### Multivariate associations with selective attention

In the multiple linear regression model presented in Table 5, soil-transmitted helminth infection was statistically significantly and negatively associated with the mean CP score. The mean CP score of children with soil-transmitted helminth infection was 7.99 points lower compared to their non-infected peers. Grip strength and the estimated mean VO_2_ max were statistically significantly and positively associated with the mean CP score. The mean CP score increased by 0.98 points per ml kg^-1^ min^-1^ VO_2_ max, whereas the mean CP score increased by 0.92 points per kg grip strength. Age and soil-transmitted helminth infection were negatively associated with the error percentage in the d2 test of attention. The mean error percentage increased by 1.6% per year of age whereas a soil-transmitted helminth infection was associated with a 3.3% higher error percentage compared to non-infected children. The mean VO_2_ max was statistically significantly and positively associated with the mean E% score. The mean E% score decreased by 0.24% per ml kg^-1^ min^-1^ VO_2_ max.

**Table 5:** Demography, socioeconomic status, helminth infection, nutrition, and physical fitness as risk factors for selective attention and academic performance.

**Mean concentration (CP)**	Multiple linear regression		
Explanatory variables	B	95% CI	*p*-value^i^
Socioeconomic status (in points 0–9)^a^	0.19	-0.87 to 1.25	0.725
Age (in years)	-1.91	-4.62 to 0.80	0.166
**Sex (reference: male)**^b^	5.47	1.05 to 9.89	**0.015**
**Helminth infection (reference: uninfected)**^c^	-7.99	-14.15 to -1.84	**0.011**
Stunting (reference: non-stunted)^d^	-0.35	-2.70 to 2.01	0.711
Food insecurity^e^	1.40	-0.74 to 3.54	0.201
**VO**_**2**_ **max (in ml kg**^**-1**^ **min**^**-1**^**)**^f^	0.98	0.47 to 1.50	**<0.001**
**Grip strength (in kg)**^g^	0.92	0.08 to 1.77	**0.032**
**Mean error percentage (E%)**	Multiple linear regression		
Explanatory variables	B	95% CI	*p*-value^i^
Socioeconomic status (in points 0–9)^a^	-0.05	-0.51 to 0.40	0.813
**Age (in years)**	1.58	0.42 to 2.74	**0.008**
Sex (reference: male)^b^	0.14	-1.76 to 2.04	0.889
**Helminth infection (reference: uninfected)**^c^	3.27	0.75 to 5.79	**0.011**
Stunting (reference: non-stunted)^d^	0.07	-0.94 to 1.08	0.893
Food insecurity^e^	-0.57	-1.49 to 0.35	0.226
**VO**_**2**_ **max (in ml kg**^**-1**^ **min**^**-1**^**)**^f^	-0.24	-0.46 to -0.02	**0.035**
Grip strength (in kg)^g^	-0.19	-0.55 to 0.18	0.069
**Academic achievement scores**^h^	Multiple linear regression		
Explanatory variables	B	95% CI	*p*-value^i^
**Socioeconomic status (in points 0–9)**^a^	0.06	0.14 to 0.10	**0.008**
**Age (in years)**	-0.43	-0.54 to -0.33	**<0.001**
**Sex (reference: male)**^b^	0.42	0.25 to 0.60	**<0.001**
**Helminth infection (reference: uninfected)**^c^	-0.45	-0.72 to -0.18	**0.001**
Stunting (reference: non-stunted)^d^	0.01	-0.09 to 0.10	0.914
Food security^e^	0.07	-0.02 to 0.15	0.123
**VO**_**2**_ **max (in ml kg**^**-1**^ **min**^**-1**^**)**^f^	0.02	-0.00 to 0.04	**0.032**
Grip strength (in kg)^g^	0.02	-0.02 to 0.05	0.370

*Notes*. Statistically significant variables are marked in bold.

^a^SES: Socioeconomic status measured by ownership and housing-related questions on a scale from 0 to 9 points (0 = low score).

^b^Sex reference (male = 1, female = 2).

^c^Helminth infection: infected with one or two soil-transmitted helminth species (*A*. *lumbricoides* and/or *T*. *trichiura*) (0 = uninfected, 1 = infected).

^d^Stunted is defined as: <-2HAZ (0 = non-stunted, 1 = stunted).

^e^Food security: measured with the hunger scale ranging from 0 to 4 (0 = hungry/food insecure, 4 = not hungry/food secure).

^f^All mean VO_2_ max estimates are expressed in ml kg^-1^ min^-1^ and are adjusted for age.

^g^All mean grip strength values are expressed in kg and are not adjusted for age.

^h^Academic achievement scores: Average of the four subjects Home Language, Additional Language, Mathematics, and Life Skills (n = 777).

^i^All *p*-values are calculated using multiple linear regression, and are adjusted for clustering within school classes.

### Multivariate associations with academic achievement

In the multiple linear regression model, lower SES, male sex, higher age, being infected with soil-transmitted helminths, and a lower cardiorespiratory fitness were statistically significantly and negatively associated with academic achievement. The mean academic achievement score increased by 0.06 per point in the SES score. By contrast, children’s academic achievement score decreased by 0.43 per additional year of age and was 0.45 lower among children classified as being infected with soil-transmitted helminths compared to non-infected peers. Boys had 0.42 lower academic achievement scores than girls and a higher VO_2_ max was associated with higher academic achievement, yet only with a marginal increase of 0.02 per ml kg^-1^ min^-1^ VO_2_ max. No significant associations were observed for stunting, food insecurity, and grip strength.

## Discussion

The most important findings of the present study are that, in the multivariate analyses, soil-transmitted helminth infections and lower physical fitness were negatively associated with selective attention, while lower SES, positive soil-transmitted helminth infection status, lower cardiorespiratory fitness, and higher age were associated with poorer academic achievement. Without implying causality, our data suggest that an infection with *T*. *trichiura*, *A*. *lumbricoides*, or both, is associated with lower selective attention capacity (in terms of attention capacity and accuracy) and reduced physical fitness among school-aged children in terms of muscular strength measured as grip strength. Moreover, children infected with soil-transmitted helminths had significantly lower academic achievement scores. It is conceivable that the general well-being of infected children, as expressed in abdominal pain, fatigue, and listlessness, negatively affects their cognitive performance [21,25]. In a study by Liu et al. [22] carried out in South-western China, children infected with either *T*. *trichiura* or *A*. *lumbricoides* were also lagging behind their non-infected peers. In the same study from China, infection with one or multiple species of soil-transmitted helminths was associated with reduced speed of processing and working memory performance and worse school performance (in terms of standardized mathematics test scores).

Heavy *A*. *lumbricoides* and *T*. *trichiura* infections have been associated with cognitive impairment and were both linked with significantly increased disability weight (DW) in the Global Burden of Disease (GBD) study [45]. Our finding that soil-transmitted helminths are associated with reduced attention capacity and accuracy is novel and warrants further investigation. Yet, to our knowledge, there is no conclusive evidence whether reduced physical fitness and strength are a direct consequence of soil-transmitted helminth infection. Our analyses did not reveal any associations between VO_2_ max and single or double species helminth infections. Müller et al. [46] found that 9-year-old boys infected with *T*. *trichiura* had a lower mean VO_2_ max estimate in a slightly different sample of children from the same cohort. Of note, another cross-sectional study by Müller et al. [47] did not find any correlation between VO_2_ max results and soil-transmitted helminth infections among school-aged children from Côte d’Ivoire, which is at odds with findings from China by Yap et al. [48] who reported reduced VO_2_ max estimates of school-aged children infected with *T*. *trichiura*. In our study, irrespective of age, children infected with *A*. *lumbricoides*, *T*. *trichiura*, or both species concurrently had a lower mean grip strength compared to non-infected children. Yap et al. [20] reported increased grip strength one month after albendazole treatment. Given these findings, further research is needed to deepen the understanding of whether and how soil-transmitted helminth infections are related to VO_2_ max and grip strength among school-aged children.

The univariate analyses also suggested that stunted children have deficits in selective attention and achieve lower academic performance compared to non-stunted children. However, these associations disappeared in the multiple regression analyses. Thus, while previous research suggested that the main causes of stunting include intrauterine growth retardation, inadequate nutrition, and poor dietary diversity to support the rapid growth and development of infants and young children [49], and that stunting can result in cognitive impairments [49,50], the association between stunting and the outcomes was no longer significant after all possible influences were taken into account. In the present study, multivariate analyses are warranted as some of the independent variables were associated. For instance, our findings confirmed that stunted children had a significantly higher risk of being infected with soil-transmitted helminth, which is in line with prior research showing that chronic soil-transmitted helminthiasis is a cause of stunting [49].

The univariate analyses further showed that stunted and non-stunted children differed significantly in grip strength, whereas they had similar mean VO_2_ max values. Our findings align with a study of Malina et al. [51] reporting that stunted children had lower grip strength than their non-stunted peers. Grip strength was shown to be a valid indicator for total muscle strength in children [52], and was associated with physical health outcomes in previous studies with children and adolescents [53]. While we did not observe a statistically significant difference, a recent study by Armstrong et al. [54] found that stunted South African primary school children also performed poorer in a 20 m shuttle run test as well as in other physical fitness tests, a finding corroborated by Yap et al. [48] who reported a lower mean VO_2_ max estimate of stunted school children in China.

With regard to selective attention and academic achievement and how they might be associated with soil-transmitted helminth infection status, stunting, food insecurity, and physical fitness, we found that attention capacity is associated with infection status and physical fitness. This confirms the notion of a negative relationship between *T*. *trichiura* and *A*. *lumbricoides* on the one hand and cognition on the other, as reported in prior research [21,22,25,55]. Furthermore, our findings suggest that after controlling for confounding factors, academic achievement is negatively associated with age and soil-transmitted helminth infection, and positively associated with SES.

Only few studies have looked at the relationship between children’s physical fitness and their selective attention in low socioeconomic settings. A study by Tine and Butler [56] reported improvements in selective attention after a 12 min session of aerobic exercise in both lower- and higher-income children. Lower-income children exhibited greater improvements in selective attention compared to their higher income peers. The fact that aerobic fitness was associated with selective attention in our sample of disadvantaged school children, combined with the finding of Tine et al. [56] is highly encouraging since (i) primary school children’s aerobic fitness can be improved through regular training [57], and (ii) selective attention is associated with academic and cognitive outcomes [2]. As highlighted by Armstrong et al. [58], there is a particularly pronounced need for encouraging fitness in South African primary schools. However, the multifactorial nature of physical fitness and attention capacity of children growing up in socioeconomically deprived environments requires that health conditions such as asthma, fetal alcohol syndrome, and human immunodeficiency virus (HIV) infection status, which were not assessed in the present study, must also be considered [59].

Stratification by age revealed that 8- and 9-year-old children achieved better academic achievement scores than their 10- to 12-year-old peers. This may be explained by the fact that disadvantaged communities do not have the financial means to promote children with special needs or learning disabilities [59]. Children suffering from reading difficulties, attention deficit hyperactivity disorder (ADHD), fetal alcohol syndrome or neglect do not get the required academic support and as a consequence are not able to keep up with their peers. Students failing to achieve adequate grades are retained up to 3 years until they get too old and automatically progress to the next grade [59,60], which explains the wide age range of the participants in the current study. Girls seemed to achieve better academic results compared to boys, while there was no statistically significant difference between sex in the test of attention. A meta-analysis by Voyer and Voyer [61] found a consistent female advantage in school marks for all course content areas.

The present study expands previous research in several important ways; to our knowledge, associations between selective attention and soil-transmitted helminth infection status as well as stunting has not previously been investigated. It also contributes to the finding that chronic soil-transmitted helminth infections and cognitive impairment are associated [62]. Furthermore, this study provides new evidence that physical fitness might be associated with increased selective attention in children from a low socioeconomic environment, even after controlling for major covariates.

Our study has several limitations. First, our results are derived from a cross-sectional study and causal inferences cannot be drawn. Second, academic achievement was measured with the average end-of-year mark (achieved at the end of grade 3), which corresponds to the summary of four subjects (mathematics, home language, additional language, and life skills). While the objectivity of school grades can be questioned as a reliable outcome in empirical research (e.g., due to attributions or stereotypes of the teachers, different standards between classes/schools), this measure has a high ecological validity because sufficiently high grades are needed for academic promotion. Moreover, the influence of class was controlled for, and our study showed that selective attention and the academic achievement scores were moderately correlated (*r*>0.30). Third, we used an indirect measurement of VO_2_ max to assess aerobic fitness and it is still debated whether the maximal oxygen uptake is receptive enough for change [63] due to varying personal living conditions. However, this test was chosen because it seemed well suited for a resource-constrained setting due to its ease of application [36]. Furthermore, the 20 m shuttle run test proved to be a valid measure of children’s physical fitness in previous studies [64], and could be related to various health outcomes in school-aged children [65]. Fourth, anthropometric measurements were taken only once, which could be a source of increased measurement error. Fifth, only a single stool sample was obtained from each child. Hence, some of the helminth infections, particularly those of light intensity, were missed. Finally, we acknowledge that our study took place in disadvantaged communities (quintile three schools). As a consequence, variation in SES was limited, which might have resulted in an underestimation of SES as a predictor of selective attention and academic achievement.

In conclusion, our study provides new insights into the relative importance of different determinants of school children’s selective attention in a disadvantaged setting of South Africa. We found that soil-transmitted helminth infection and lower physical fitness may hamper children’s capacity to pay attention during cognitive tasks, and directly or indirectly impede their academic performance. It is conceivable that poor academic achievement will hinder children from realizing their full potential and disrupt the vicious cycle of poverty and ill health.

## Supporting information

S1 Checklist STROBE checklist. (DOCX)
